# Variability of insulin sensitivity during the first 4 days of critical illness

**DOI:** 10.1186/cc10774

**Published:** 2012-03-20

**Authors:** C Pretty, A Le Compte, JG Chase, G Shaw, JC Preiser, S Penning, T Desaive

**Affiliations:** 1 University of Canterbury, Christchurch, New Zealand; 2 Christchurch Hospital, Christchurch, New Zealand; 3 Hospital Erasme, Free University of Brussels, Belgium; 4 University of Liege, Belgium

## Introduction

Safe, effective tight glycaemic control (TGC) can improve outcomes in critical care patients, but is difficult to achieve consistently. Insulin sensitivity defines the metabolic balance between insulin concentration and insulin-mediated glucose disposal. Hence, variability of insulin sensitivity can cause variable glycaemia. This study investigates the evolution of insulin sensitivity level and variability in patients receiving TGC during the first 4 days of their ICU stay.

## Methods

A retrospective analysis of patient data (*n *= 164 patients) from the SPRINT TGC study in the Christchurch Hospital ICU [1]. All patients commenced TGC within 12 hours of ICU admission and spent at least 24 hours on the SPRINT protocol. Model-based insulin sensitivity (SI) was identified using a validated glucose-insulin system model developed for critical care patients. SI was identified every hour for each patient using clinical data and the model. Level and hour-to-hour percentage changes in SI were assessed on cohort and per-patient bases. Level and variability of SI were compared over time on 24-hour and 6-hour timescales for the first 4 days of the ICU stay.

## Results

Cohort and per-patient median SI levels increased by 34% and 33% (*P *< 0.001) between days 1 and 2 of the ICU stay. Concomitantly, cohort and per-patient SI variability reduced by 32% and 36% (*P *< 0.001). For 72% of the cohort, median SI on day 2 was higher than day 1. The day 1 and 2 results were the only clear, statistically significant trends across both analyses. Analysis of the first 24 hours using 6-hour blocks of SI data showed that most of the improvement in insulin sensitivity level and variability seen between days 1 and 2 occurred during the first 12 to 18 hours of day 1. This rapid improvement was probably due to the decline of counterregulatory hormones as the acute phase of critical illness progressed.

## Conclusion

ICU patients have significantly lower and more variable insulin sensitivity on day 1 than later in their ICU stay and particularly during the first 12 hours. Clinically, these results suggest that while using TGC protocols with patients during their first few days of ICU stay, extra care should be afforded. Increased measurement frequency, higher target glycaemic bands, conservative insulin dosing and modulation of carbohydrate nutrition should be considered to safely minimize outcome glycaemic variability and reduce the risk of hypoglycaemia.
